# Rare Variants in the *MECP2* Gene in Girls with Central Precocious Puberty

**DOI:** 10.1016/S2213-8587(23)00131-6

**Published:** 2023-06-26

**Authors:** Ana P. M. Canton, Flávia R. Tinano, Leonardo Guasti, Luciana R. Montenegro, Fiona Ryan, Deborah Shears, Maria Edna de Melo, Larissa G. Gomes, Mariana P. Piana, Raja Brauner, Rafael E. Aguilar, Arancha Escribano-Munôz, Alyssa Paganoni, Jordan Read, Márta Korbonits, Carlos E. Seraphim, Silvia S. Costa, Ana Cristina Krepischi, Alexander A. L. Jorge, Alessia David, Lena R. Kaisinger, Ken K. Ong, John R. B. Perry, Ana Paula Abreu, Ursula B. Kaiser, Jesús Argente, Berenice B. Mendonca, Vinicius N. Brito, Sasha R. Howard, Ana Claudia Latronico

**Affiliations:** 1 Developmental Endocrinology Unit, Laboratory of Hormones and Molecular Genetics, LIM/42, Discipline of Endocrinology and Metabolism, Clinicas Hospital, School of Medicine, University of Sao Paulo, 05403-000, Sao Paulo, Brazil; 2 Centre for Endocrinology, William Harvey Research Institute, Barts and the London School of Medicine and Dentistry, Queen Mary University of London, London, EC1M6BQ, UK; 3 Oxford Children’s Hospital, Oxford University Hospitals NHS Foundation Trust, Oxford, UK; 4 Oxford Centre for Genomic Medicine, Oxford University Hospitals NHS Foundation Trust, Oxford, UK; 5 League of Childhood Obesity, Discipline of Endocrinology and Metabolism, Clinicas Hospital, School of Medicine, University of Sao Paulo, 05403-000, Sao Paulo, Brazil; 6 Children’s State Hospital of Vila Velha, 29106-150, Vila Velha, Brazil; 7 Fondation Ophtalmologique Adolphe de Rothschild and Université de Paris, Paris, France; 8 Hospital Universitario Virgen de Valmes, Universidad de Sevilla, 41013, Sevilla, Spain; 9 Endocrinology Unit, Department of Pediatrics, Virgen de la Arrixaca University Hospital, Murcia, Spain; 10 Department of Genetics and Evolutionary Biology, Institute of Biosciences, University of Sao Paulo, Sao Paulo, Brazil; 11 Genetic Endocrinology Unit, LIM25, Discipline of Endocrinology and Metabolism, Clinicas Hospital, School of Medicine, University of Sao Paulo, 01246-903, Sao Paulo, Brazil; 12 Centre for Integrative Systems Biology and Bioinformatics, Department of Life Sciences, Imperial College London, London, UK; 13 MRC Epidemiology Unit, Wellcome-MRC Institute of Metabolic Science, University of Cambridge School of Clinical Medicine, Cambridge CB2 0QQ, UK; 14 Division of Endocrinology, Diabetes and Hypertension, Brigham and Women’s Hospital and Harvard Medical School, 02115, Boston, USA; 15 Department of Pediatrics, Universidad Autónoma de Madrid. Department of Pediatrics and Pediatric Endocrinology, Hospital Infantil Universitario Niño Jesús, Instituto de Investigación La Princesa, Centro de Investigación Biomédica en Red de Fisiopatología de la Obesidad y Nutrición (CIBEROBN), Instituto de Salud Carlos III, IMDEA Food Institute. Madrid, Spain; 16 Department of Paediatric Endocrinology, Barts Health NHS Trust, London, UK

**Keywords:** central precocious puberty, MECP2, X-linked precocious puberty, genetics of puberty

## Abstract

**Background:**

Identification of genetic causes of central precocious puberty (CPP) has revealed epigenetic mechanisms as regulators of human pubertal timing. Methyl-CpG-binding protein 2 (*MECP2*), an X-linked gene, encodes a chromatin-associated protein with a role in gene transcription. *MECP2* loss-of-function mutations usually cause Rett syndrome, a severe neurodevelopmental disorder. Early pubertal development was demonstrated in several patients with Rett syndrome.

**Methods:**

We investigated a multiethnic cohort of 404 patients (383 girls) with idiopathic CPP for potentially damaging variants in *MECP2*, evaluating whether MECP2 might contribute to CPP etiology. We performed high-throughput sequencing in 133 patients and Sanger sequencing of *MECP2* in further 271 patients. Mice hypothalamic expression of Mecp2 and colocalization with GnRH neurons were determined.

**Findings:**

We identified three rare heterozygous likely damaging coding variants in *MECP2* in five girls: a *de novo* missense variant (p.Arg97Cys) in two monozygotic twin sisters with CPP and microcephaly; a *de novo* missense variant (p.Ser176Arg) in one girl with sporadic CPP, obesity and autism; and an insertion (p.Ala6_Ala8dup) in two unrelated girls with sporadic CPP. Additionally, we identified one rare heterozygous 3 ’UTR *MECP2* insertion (c.*36_*37insT) in two unrelated girls with sporadic CPP. None of them manifested Rett syndrome. Mecp2 protein co-localized with GnRH expression in mice hypothalamic nuclei key for GnRH regulation.

**Interpretation:**

Rare *MECP2* variants were demonstrated in girls with idiopathic CPP, with or without mild neurodevelopmental abnormalities. MECP2 may have a role in the hypothalamic control of human pubertal timing, increasing evidence of (epi)genetic mechanisms in this biological process.

## Introduction

Central precocious puberty (CPP) is defined by the premature development of secondary sexual characteristics due to the early reactivation of pulsatile hypothalamic gonadotropin-releasing hormone (GnRH) secretion. Characteristically, the overall frequency of CPP is much higher in girls than in boys (1). The recognition of genetic causes underlying CPP has increased mainly through high-throughput sequencing studies of familial forms of CPP (2, 3). To date, loss-of-function mutations in two autosomal imprinted genes [Makorin ring finger protein 3 (*MKRN3*) and Delta like non-canonical Notch ligand 1 (*DLK1*)] are the most prevalent known monogenic causes of familial CPP with the phenotype exclusively associated with paternal transmission. The pathogenic mechanisms of deficiency of both genes involve epigenetic regulation of upstream pathways of GnRH neuronal activity (2, 3, 4). The etiological diagnosis of CPP may benefit individuals through a precision medicine approach, providing patients personalized strategies of follow-up with increased long-term clinical surveillance and allowing genetic counselling within families (4).

X-linked gene defects have long been considered important causes of central nervous system (CNS) disorders, since these genes are highly expressed in the brain (5). The potential role of X-linked genes in human pubertal development has been suggested by distinct lines of evidence, such as descriptions of early puberty in patients harboring X-chromosome structural variants, enrichment of X-linked differentially methylated regions in methylome profiling of girls at puberty, and genomic association studies in large populations (6, 7). Most human X-linked genes are subject to X-inactivation in females, a mechanism that ensures dosage compensation between both sexes (5, 8).

Methyl-CpG-binding protein 2 (*MECP2*) is a X-linked gene (chromosome Xq28) which encodes a nuclear protein capable of binding to methylated DNA in promoter regions, functioning as a repressor or an activator of gene transcription (9, 10). *MECP2* is widely expressed in human tissues, with the highest expression observed in the brain (9). Loss-of-function mutations in *MECP2* are usually associated with neurodevelopmental disorders, in particular with Rett syndrome (11). Rett syndrome (OMIM#312750) is a severe neurodevelopmental disorder characterized by the regression of acquired skills between age 12 and 30 months. Its main diagnostic criteria are partial or complete loss of acquired purposeful hand skills and acquired spoken language, absent or abnormal gait, and stereotypic movements principally involving the hands (12). Additional comorbidities include epilepsy, scoliosis, and gastrointestinal dysfunction. *MECP2* mutations in Rett patients are most commonly due to *de novo* mutations that arise on the paternal X-chromosome (13). However, broader clinical phenotypes have been described in individuals with *MECP2* mutations, such as mild neuro-disabilities characterized by autistic spectrum disorder or intellectual impairment, suggesting that distinct *MECP2* variants may lead to diverse phenotypic consequences (12, 14).

Notably, early pubertal development has been documented among children with Rett syndrome due to *MECP2* mutations (15, 16). An observational American study described early thelarche in 25% and early pubarche in 28% of Rett girls; furthermore, menarche occurred earlier in those girls with milder mutations (15). Similarly, mean age of thelarche was 7.1 years in a larger Rett cohort (earlier than the mean age in healthy Caucasian girls) (16). Mean age of menarche was within the normal range in most Rett girls from both studies, leading to a longer time from thelarche to menarche. Nevertheless, CPP was diagnosed in case reports of Rett patients with *MECP2* mutations (17). In this original study, we explored whether *MECP2* variants are associated with an idiopathic CPP phenotype, evaluating its potential role in the etiology of this endocrine disorder.

## Methods

### Patients

In total, a multiethnic cohort of 404 patients with idiopathic CPP (383 girls and 21 boys; 143 familial cases from 134 unrelated families and 261 sporadic cases) were investigated for rare potentially damaging variants in the *MECP2* gene. The diagnosis of CPP was defined as the development of progressive pubertal signs, primarily Tanner stage 2 breast development and testicular volume before age 8 years in girls and 9 years in boys, respectively. Basal and/or GnRH-stimulated luteinizing hormone (LH) levels within the pubertal range confirmed the diagnosis of CPP (1). In females, precocious menarche (≥ 9 years) was considered an equivalent clinical signal of CPP. None of the patients had significant anatomical abnormalities related to CPP on magnetic resonance imaging of the CNS. All patients were clinically assessed by pediatric endocrinologists with expertise in genetic disorders. Detailed criteria of patients are described in Supplementary Appendix. The study protocol was approved by the respective local Ethics Committees. Written informed consent was obtained from all patients and their legal guardians.

### Genetic studies

Firstly, high-throughput sequencing studies were performed in 133 CPP patients, as part of independent genetic investigations based on multigene sequencing approaches at Sao Paulo University (n=111) and Queen Mary University of London (n=22). Among these, whole-exome sequencing was performed in 62 patients (7 sporadic and 55 familial cases), while targeted gene sequencing (panel of 746 genes, including *MECP2*) was performed in 71 patients (54 sporadic and 17 familial cases). Subsequently, the *MECP2* gene was screened by Sanger sequencing in 271 additional CPP patients to expand the analysis of *MECP2* in a larger cohort. Studies to determine parental origin of variants were performed in patients carrying *de novo MECP2* variants, by analysis of linkage between the *MECP2* variants and nearby heterozygous inherited polymorphisms. X-inactivation analyses of blood samples (leukocyte DNA) were performed in girls carrying *MECP2* variants and their mothers.

All the methods employed were undertaken as previously described and were conducted according to the standard protocol of the manufacturer (6, 18). The genomic positions and the raw data of all experiments were aligned using the GRCh38/hg38 assembly of the human genome reference. All possible candidate variants were classified according to the American College of Medical Genetics and Genomics (ACMG) standards with five categories of pathogenicity: pathogenic, likely pathogenic, variant of uncertain significance (VUS), likely benign, and benign. To provide further genetic evidence of the association of *MECP2* with CPP phenotype, an analysis of rare potentially damaging variants across the entire gene was performed, comparing allele frequencies between the CPP cohort (cases) and the Genome Aggregation Database (gnomAD; https://gnomad.broadinstitute.org) public database (controls). The samples from the CPP cohort and the gnomAD database were not jointly sequenced or called. Allele frequency differences between groups were analyzed by Fisher’s exact test. Statistical significance was set at *p*-value <0.05. Detailed methods are described in the Supplementary Appendix.

### Assays in mice

Immunochemistry studies were conducted from hypothalamic tissue sections of pubertal (postnatal day 38) female mice collected from timed crosses of C57BL/6 mice. Anti-MECP2 and anti-GnRH primary antibodies were used for tissue expression analysis. Detailed analyses are described in the Supplementary Appendix.

## Role of the funding source

The funders of the study had no role in the study design, data collection, data analysis, data interpretation, or writing of the report.

## Results

### MECP2 rare variants identified in CPP patients

**Figure 1:**
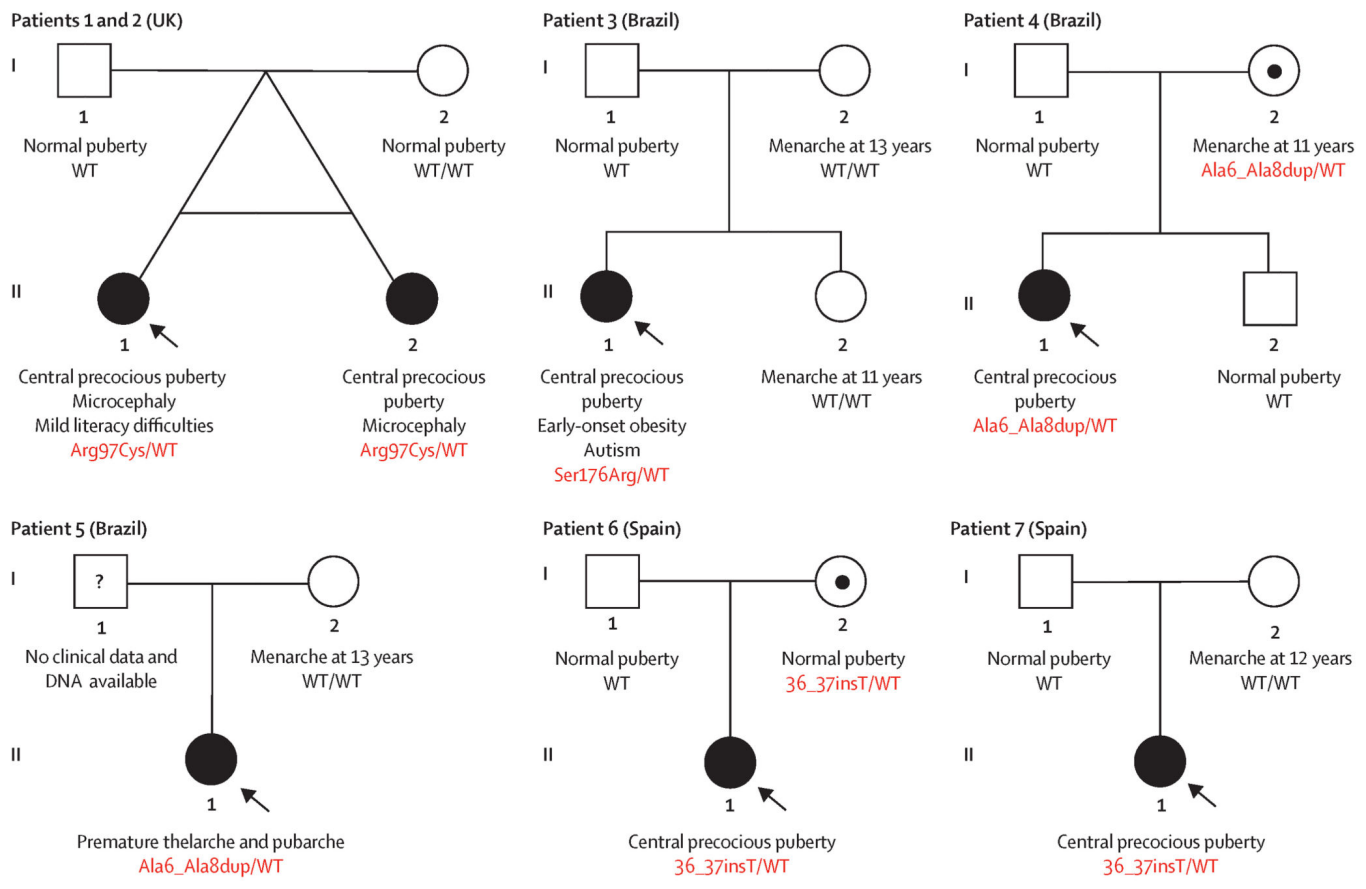
Pedigrees of patients with central precocious puberty associated with rare heterozygous *MECP2* variants. Squares indicate male family members, circles female family members, black symbols clinically affected family members, symbols with black internal circles asymptomatic carriers, symbols with a question mark family member whose phenotype is unknown and arrows the proband in each family. Pubertal characteristics are shown for each individual; neurodevelopmental disorders are shown, when identified. The *MECP2* genotype is shown for individuals whose DNA was available for genetic studies. Wt denotes wild-type genotype.

**Figure 2:**
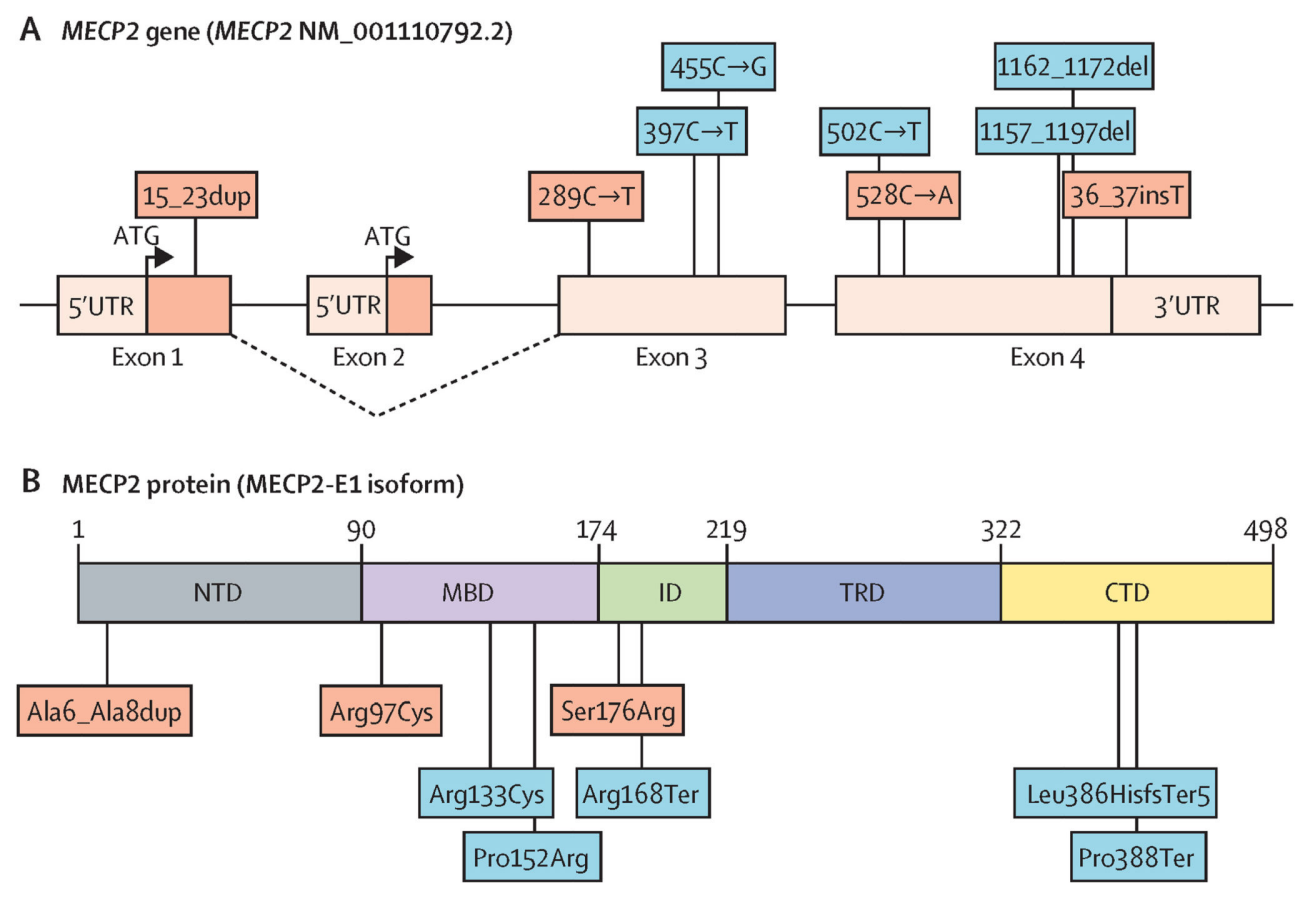
Schematic representation of the *MECP2* gene structure (four exons) and the MECP2 protein (six protein domains). The MECP2-E1 isoform is derived from alternate splicing of exon 2 (shown in dashed lines), and it corresponds to the NM_001110792.2 transcript. In the current study, four heterozygous *MECP2* variants (shown in red) were identified in seven girls with central precocious puberty (CPP). Among them, three CPP girls had missense mutations (two in the methyl-binding domain and one in the intervening domain), while four girls had *MECP2* insertions. In previous studies, five heterozygous *MECP2* variants (shown in blue) were identified in six girls with Rett syndrome diagnosed with CPP (17). Among them, two Rett girls with CPP had missense mutations in the methyl-binding domain, while four girls had truncating mutations (one in the intervening domain and three in the C-terminal domain). NTD: amino-terminal domain; MBD: methyl-binding domain; ID: intervening domain; TRD: transcriptional repression domain; CTD: carboxi-terminal domain.

We identified four rare heterozygous *MECP2* variants in seven girls with CPP (Table 1 and Figure 1). Three *MECP2* variants located at the coding region were identified in five girls (from four unrelated families) by high-throughput sequencing analysis, and all were predicted to be damaging by several *in silico* tools, including a function-structure analysis (Figure 2). Additionally, a *MECP2* insertion located at the 3’ untranslated region (UTR) was identified in two unrelated girls by Sanger sequencing analysis. Among the seven affected girls, *MECP2* variants were classified as likely pathogenic in three patients and as VUS in four patients by ACMG criteria. All variants were mapped to the *MECP2* transcript NM_001110792.2. Detailed genetic findings are described in Supplementary Appendix.

Two British monozygotic twin sisters (Patients 1 and 2) with CPP carried a heterozygous missense variant in exon 2 (c.289C>T) of *MECP2*, corresponding to the methyl-CpG-binding domain (MBD) of the protein (p.Arg97Cys) (19). The p.Arg97Cys variant was predicted to be damaging in 19 of 21 *in silico* programs (Supplementary Appendix) and identified at a very low allelic frequency in gnomAD (1/182,926). The position of this variant might be implicated in stabilizing the interaction between MECP2 and DNA. Familial segregation analysis revealed that the p.Arg97Cys variant was a *de novo MECP2* mutation. Biological paternity was confirmed.

One Brazilian girl with sporadic CPP (Patient 3) carried a heterozygous missense variant in exon 3 (c.528C>A) of *MECP2*, corresponding to the intervening domain of the protein (p.Ser176Arg) (19). The p.Ser176Arg variant was predicted to be damaging by 15 of 20 *in silico* programs (Supplementary Appendix) and absent in Online Archives of Brazilian Mutations (ABraOM; https://abraom.ib.usp.br) and gnomAD. The serine at this position was evolutionarily conserved and phosphorylation at this residue highly impaired MECP2 binding to DNA or chromatin *in vitro* (20). Familial segregation analysis revealed that the p.Ser176Arg variant was a *de novo MECP2* mutation. Biological paternity was confirmed. Therefore, both missense variants p.Arg97Cys (Patients 1 and 2) and p.Ser176Arg (Patient 3) were predicted to interfere with protein function and were classified as likely pathogenic.

Two unrelated Brazilian girls with sporadic CPP (Patients 4 and 5) carried a heterozygous *indel* insertion of 9 bp in exon 1 (c.15_23dup) of *MECP2*, corresponding to the N-terminal domain of the protein (p.Ala6_Ala8dup) (19). The insertion was within an amino acid sequence specific to the NM_001110792.2 transcript (21). It was located at a highly conserved region, consisting of a polyalanine tract. Previous studies suggested that expansion of alanine repeats could alter protein subcellular localization, which could cause retention of MECP2 within the cytoplasm (22). This insertion was absent in ABraOM and identified at a very low allelic frequency in gnomAD (3/59,976). Familial segregation analysis revealed that Patient 4 inherited the p.Ala6_Ala8dup variant from her unaffected mother. Meanwhile, it was absent in the mother of Patient 5; clinical data and DNA from her father were not available. Therefore, the p.Ala6_Ala8dup variant was classified as of uncertain significance with major pathogenic evidence.

Two unrelated Spanish girls with sporadic CPP (Patients 6 and 7) carried a heterozygous insertion (c.*36_*37insT) in the 3’UTR of *MECP2*. This insertion was absent in ABraOM and gnomAD and it was in a region that is highly conserved across mammals. Previous studies demonstrated that the *MECP2* 3’UTR harbors multiple polyadenylation sites and miRNA binding sites (23). Familial segregation analysis revealed that Patient 6 inherited the insertion from her unaffected mother and Patient 7 had a *de novo* insertion with biological paternity confirmed. The c.*36_*37insT variant was classified as of uncertain significance.

We studied the parental origin of the *de novo MECP2* variants (Patients 1, 2, 3, and 7). Based on the presence of an informative heterozygous polymorphism (rs3027928), we could determine that p.Ser176Arg variant arose on the paternal X-chromosome in Patient 3. The remaining patients had no informative polymorphisms near the *de novo MECP2* variants to specifically determine the parental origin.

According to available databases, these four rare *MECP2* variants identified in CPP girls have not been associated with Rett syndrome phenotype, except for one case with the p.Arg97Cys variant (https://www.ncbi.nlm.nih.gov/clinvar/variation/424578/?oq=rs1064797047&m=NM_001110792.2(MECP2):c.289C%3ET%20(p.Arg97Cys).

The allele frequency of rare potentially damaging *MECP2* variants identified in CPP girls (0.91%) was significantly higher than that identified in females from the gnomAD database (0.09%; unadjusted *p*=1.587e-05; Table 3).

We evaluated the impact of *MECP2* variants in UK Biobank, a population study with exome sequencing and phenotypic characterization (https://www.ukbiobank.ac.uk). In 222,283 female participants with both exome sequencing data and information on recalled age at menarche, we identified 18 women carrying three *MECP2* variants described above (p.Arg97Cys variant n=1; p.Ala6_Ala8dup variant n=12; c.*36_*37insT variant n=5). The mean age at menarche of these 18 women was 13.0 ±1.57 years, not statistically different from the Biobank average (mean age at menarche 12.9 ±1.61 years; *p*=0.92). One woman carrying the p.Ala6_Ala8dup variant had menarche at age 10 years. Among male participants, five men carried three *MECP2* variants (p.Arg97Cys variant n=1; p.Ala6_Ala8dup variant n=1; c.*36_*37insT variant n=3) with an average age of voice breaking. The p.Ser176Arg variant was not identified in any individual, confirming its very rare frequency. In UK Biobank publicly available data, high-confidence protein truncating variants in *MECP2* were identified in 7 females and no males;these variants had no significant associations with age at menarche, body mass index, height, or testosterone levels. Data on signs of pubertal onset (thelarche, testicular enlargement, and pubarche) and polycystic ovary syndrome (PCOS) were not available in the Biobank.

Additionally, we evaluated *MECP2* for associations with human conditions in genomewide association studies (GWAS). To date, no locus near *MECP2* has been associated with measured pubertal traits (https://www.ebi.ac.uk/gwas/genes/MECP2).

Studies of X-inactivation pattern in blood samples of ten females (six patients and four mothers; Supplementary Table 1) identified an extreme skewing in Patient 1. This finding implied that one of the X-chromosomes was preferentially inactivated in this patient, at least in the blood. Skewed X-inactivation can be observed in a small proportion of healthy females due to stochastic processes, but also can be associated with clinical conditions caused by the presence of a pathogenic sequence variant in the X-chromosome, such as Rett syndrome due to *MECP2* mutations (24). No other extreme skewed X-inactivation was identified in blood samples of the remaining females evaluated.

### Clinical phenotypes of CPP patients with MECP2 variants

Precocious thelarche was identified by physical exam as the first sign of premature pubertal development in six (of seven) girls with *MECP2* variants (Table 2). The median age of onset of breast development was 5.4 years (ranging from 0.7 to 7.6). The remaining girl (Patient 3) came for the first endocrine visit with a report of precocious menarche at age 8.5 years. Bone age, when available, showed a typical advancement in relation to chronological age (≥ 2 years). None had family history of premature sexual development.

Patients 1, 2, and 3, who carry missense likely pathogenic *MECP2* variants (p.Arg97Cys and p.Ser176Arg, respectively), had CPP and neurobehavioral phenotypes. These three girls were submitted to a comprehensive assessment with clinical geneticists and neuro-pediatricians, which did not identify criteria for the diagnosis of a recognized syndrome, including Rett syndrome. Patients 1 and 2, the monozygotic twin sisters, exhibited microcephaly and Patient 1 had also mild difficulties in literacy, but no other neurodevelopmental phenotypes on assessment. Patient 3 had autistic spectrum disorder (confirmed by standardized neuropsychological evaluation),macrocephaly, and hyperphagia followed by early-onset obesity. In contrast, the unrelated girls carrying respectively the p.Ala6_Ala8dup insertion (Patients 4 and 5) and the c.*36_*37insT insertion (Patients 6 and 7) had no evident neurodevelopmental abnormalities. Of relevance, both girls carrying the p.Ala6_Ala8dup insertion developed irregular menstrual cycles and other findings resembling PCOS as young adults. All clinical findings are detailed in Supplementary Appendix.

### Mecp2 was expressed in key hypothalamic nuclei responsible for GnRH regulation

**Figure 3:**
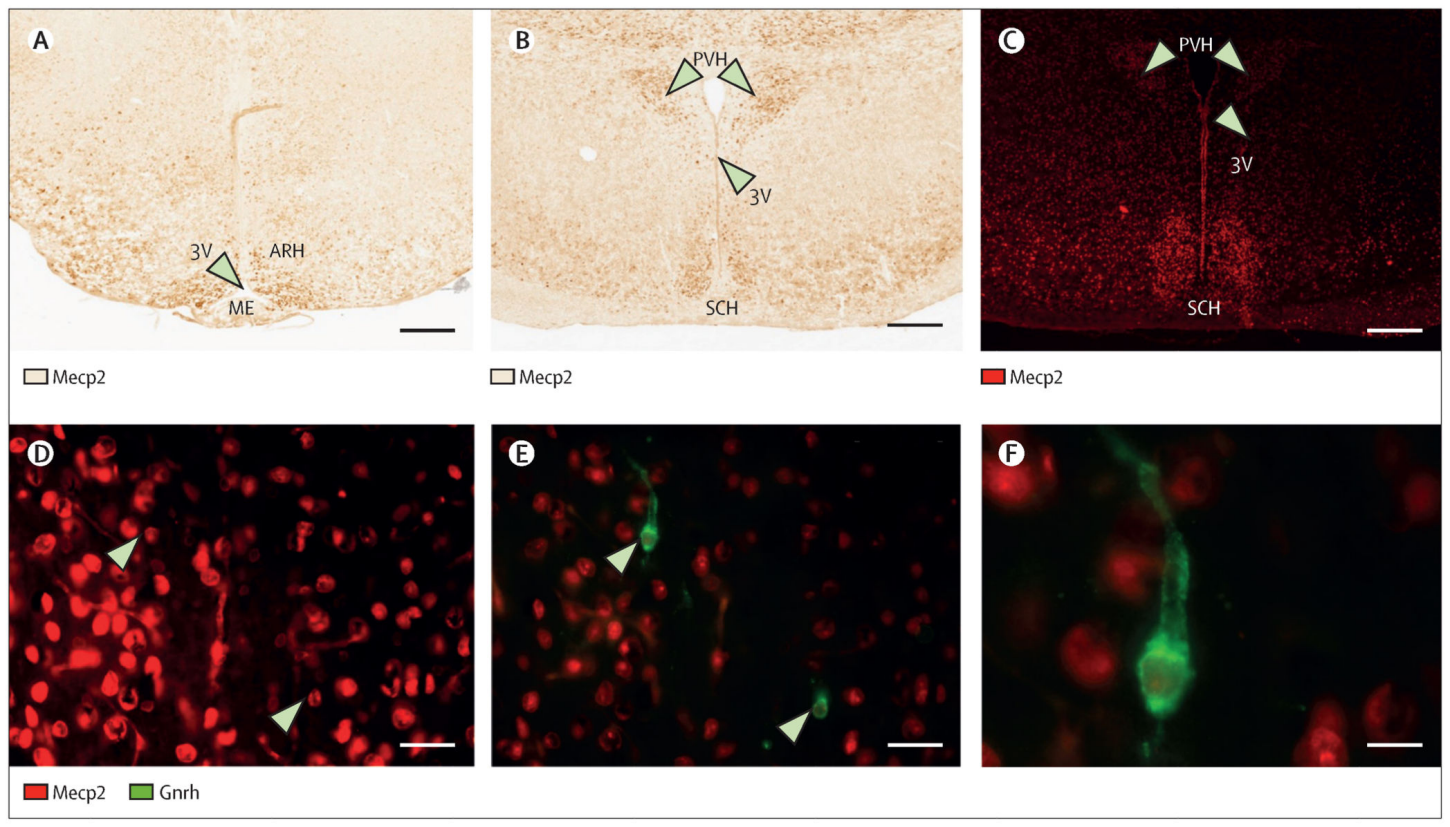
Tissue expression of Mecp2 and Gnrh in postnatal (day 38) female mouse hypothalamus. Mecp2 is expressed in key regions for GnRH neuronal function. **A)** and **B)** Immunohistochemistry analysis revealing Mecp2 localization in the arcuate and suprachiasmatic nuclei of the hypothalamus, as well as the paraventricular hypothalamic nucleus, parvicellular division and median eminence at postnatal day 38 in female mouse. **C)** and **D)** Immunofluorescent staining for Mecp2 (in Red) confirms localization to the same regions of the hypothalamus, and **E)** and **F)** is suggestive of co-localization within the paraventricular nucleus of GnRH (in Green) and Mecp2 (in Red); DAPI nucleolar staining for the same sections (in Blue). Scale bar A-D: 100 μm; E-G: 50 μm; H-J 25 μm. Representative images of experiments performed at least 3 independent times. Arrowheads point to GnRH neurons; ARC: arcuate nucleus; SCH: suprachiasmatic nucleus; PVH; paraventricular hypothalamic nucleus; 3V: third ventricle; ME median eminence; coronal sections.

Immunohistochemistry and immunofluorescence demonstrated abundant staining for Mecp2 in multiple relevant hypothalamic nuclei, including arcuate, suprachiasmatic, and paraventricular nuclei, and in the median eminence, in pubertal female mice (Figure 3). Double labelling experiments showed co-localization of Mecp2 and Gnrh in more than 70% of GnRH neuronal cells visualized.

## Discussion

Loss-of-function mutations in two maternal imprinted genes (*MKRN3* and *DLK1*) have been identified as the most frequent etiologies of familial CPP cases, indicating an essential role for DNA methylation among the mechanisms underlying pubertal timing (2, 3). Both genes are located at critical regions of imprinting disorders that may be associated with CPP (*MKRN3* at chromosome 15q11-q13 of Prader-Willi syndrome and *DLK1* at chromosome 14q32.2 of Temple syndrome) (6). In this original study, we have demonstrated a potential X-linked form of CPP associated with rare variants in *MECP2*, a key component of the human DNA methylation machinery, whose inactivation has previously been associated with Rett syndrome (25). We identified seven girls (from six unrelated families) with CPP carrying four rare heterozygous *MECP2* variants (p.Arg97Cys, p. Ser176Arg, p.Ala6_Ala8dup, and c.*36_*37insT). None of these girls with CPP and *MECP2* variants manifested Rett syndrome.

The MECP2 protein belongs to the methyl-binding domain family, that in mammals are mediators of DNA methylation, a major epigenetic mechanism that occurs predominantly in the context of CpG dinucleotides (25). The known functions of MECP2 involve repression or activation of gene transcription and regulation of chromatin structure (9, 10, 25), playing a role in the epigenetic regulation of target gene expression (10). Growing evidence has shown the participation of epigenetic mechanisms in the hypothalamic control of puberty development. Recently, animal studies proposed that the Methyl-CpG-binding domain protein 3 (MBD3), another protein of the methyl-CpG-binding domain family, was a substrate for the ubiquitin activity of MKRN3. The MKRN3-MBD3 axis regulated the methylation status of *GNRH1* promoter, directly regulating pubertal timing (26).

The seven girls identified with rare heterozygous *MECP2* variants had a sporadic form of CPP. This clinical form is in line with the identification of *de novo* variants in four of these girls, a pattern considered as strong evidence for pathogenicity. In one girl (Patient 3), the *de novo* p.Ser176Arg variant was shown to arise from the paternal X-chromosome, a mechanism that may partially explain the female predominance of *MECP2* mutations (13). In two other girls, the *MECP2* variants were inherited from their unaffected mothers, a pattern of clinical variability that has been demonstrated among females with defects in genes subject to X inactivation (8, 12). Mild neurobehavioral abnormalities were identified in girls carrying *de novo* missense *MECP2* variants (Patients 1 to 3). Such cases might be considered syndromic forms of CPP and thus represent part of the spectrum of neurodevelopmental disorders related to *MECP2* (14). The girls with CPP carrying *MECP2* insertions (Patients 4 to 7) did not manifest clear neurobehavioral symptoms, suggesting milder clinical pictures. This clinical heterogeneity could be related to the severity of *MECP2* variants, where the more damaging the variant, the more complex the phenotype. Notably, the phenotypic spectrum of patients with *MECP2* mutations is highly variable, ranging from Rett syndrome to other *MECP2-related* disorders, or to unaffected carrier mothers in rare familial cases (25). This variable expressivity could be partially attributed to the tissue-specific patterns of X-chromosome inactivation that could result in distinct MECP2 expression profiles in the brain, leading to heterogeneous phenotypes observed in individuals carrying the same mutations (27). Our findings of rare heterozygous *MECP2* variants in females with CPP, including two unaffected carrier mothers, might be secondary to this phenomenon.

The association testing of rare variants demonstrated a significantly higher frequency of rare potentially damaging *MECP2* variants in CPP girls than in controls, indicating an enrichment of *MECP2* variants in this cohort and further strengthening a putative association of *MECP2* with the CPP phenotype. Although UK Biobank data did not identify an association between precocious menarche and rare damaging variants in *MECP2*, data on age at thelarche and pubarche were not available from the Biobank study. This result may suggest a stronger effect of MECP2 deficiency on pubertal onset than completion (menarche), as is observed in cohorts of female patients with Rett syndrome due to *MECP2* mutations (15, 16). In addition, the identification of females with normal mean age at menarche carrying three distinct *MECP2* variants in the Biobank also suggested a possible reduced penetrance or variable expressivity, which could be modulated by distinct patters of X-inactivation or by compensatory mechanisms. Notably, in GWAS no *locus* near *MECP2* was associated with measured pubertal traits to date, nor with neurological conditions often associated with *MECP2* mutations.

We demonstrated that Mecp2 is highly expressed in key areas of the hypothalamus responsible for GnRH function in female mice and co-localized with GnRH in most GnRH neurons in these regions. A similar result was recently demonstrated in a study showing co-expression of MECP2 with GnRH and kisspeptin in the hypothalamus of ewes (28). The exact mechanism by which *MECP2* might influence hypothalamic GnRH secretion is not yet known. A potential intermediatory factor is *FXYD1*, which expression was increased in the brain of a mouse *Mecp2* knockout model (29). Studies in rodents suggested that *Fxyd1* promoted GnRH neuronal excitability, facilitating the advent of puberty (16). These results suggested that loss of *MECP2* might lead to precocious pubertal timing potentially due to increased *FXYD1* activity.

Alternatively, *MECP2* might influence hypothalamic GnRH secretion via δ-aminobutyric acid (GABA), a majtaor inhibitory factor of GnRH secretion (1). Animal studies have demonstrated that MECP2 is critical for normal function of GABA-releasing neurons (30). Mecp2-deficient mice had disruption of the central balance between excitatory and inhibitory inputs, marked by a GABAergic pathway deficit. In this model, disruption of *MECP2* regulation might lead to precocious pubertal onset due to decreased GABAergic inhibition of GnRH secretion.

Investigation of genetic causes has been suggested in children with CPP presenting with the familial form or in association with multiple phenotypes, especially neurodevelopmental abnormalities (1). As genomic investigation is extended to larger cohorts of CPP patients, the recognition of key factors involved in the neuroendocrine control of pubertal timing is likely to increase. Whilst Rett syndrome has been associated with abnormal pubertal development, MECP2 function has not previously been linked to the timing of puberty in patients with previous diagnosis of idiopathic CPP. Our findings identifying rare heterozygous *MECP2* variants in multiple unrelated girls with CPP, with or without mild neurodevelopmental abnormalities, suggested a potential X-linked form of premature pubertal development.

## Supplementary Material

Supplementary Material

## Figures and Tables

**Table 1:** Rare heterozygous variants in *MECP2* identified in seven girls with central precocious puberty.

	*Patients with variant in the coding region*					*Patients with variant in the 3’ UTR*	
Patients ID	Monozygotic twin sisters		Patient 3	Patient 4	Patient 5	Patient 6	Patient 7
	Patient 1	Patient 2					
**Genomic position**	X:154032331		X:154031336	X:154097642		X:154097642	
**Variant type**	Missense		Missense	*Indel* insertion		3 ‘UTR insertion	
**cDNA**	c.289C>T		c.528C>A	c.15_23dup		c.*36_*37insT	
**Protein**	p.Arg97Cys		p.Ser176Arg	p.Ala6_Ala8dup		-	
**Familial segregation**	*de novo*		*de novo*	Inherited from the unaffected mother	Absent in the unaffected mother^*a*^	Inherited from the unaffected mother	*de novo*
**Population data (allele frequency)**							
**gnomAD**	0.000005467		Not reported	0.00005002		Not reported	
**ABraOM**	Not reported		Not reported	Not reported		Not reported	
**Protein domain**	Methyl-CpG-binding domain		Intervening domain	N-terminal domain		-	
**ACMG classification**	Likely pathogenic		Likely pathogenic	VUS		VUS	
**Previous association with Rett syndrome phenotype** ^*b*^	1 case in ClinVar		No	No		No	

UTR: untranslated region; gnomAD: Genome Aggregation Database; ABraOM: Online Archive of Brazilian Mutations; ACMG: American College of Medical Genetics and Genomics standards; VUS: variant of uncertain significance All sequence variants are according to the transcript identified as NM_001110792.2 by the Human Genome Variation Society (HGVS). All genomic positions are according to the GRCh38/hg38 assembly of the human genome reference.

a DNA and clinical data from the father were not available.

b Previous association with Rett syndrome phenotype was evaluated by analysis of published literature, ClinVar data, and RettBASE data.

**Table 2:** Clinical and hormonal features of seven girls with central precocious puberty and rare heterozygous variants in *MECP2*.

Patient ID	Monozygotic twin sisters		3	4	5	6	7
	1	2					
***MECP2* variant**	Missense substitution p.Arg97Cys		Missense substitution p.Ser176Arg	*Indel* insertion p.Ala6_Ala8dup	*Indel* insertion p.Ala6_Ala8dup	3 ’UTR insertion c.*36_*37insT	3 ’UTR insertion c.*36_*37insT
**Ethnicity**	European (United Kingdom)		Brazilian	Brazilian	Brazilian	European (Spain)	European (Spain)
**Age at pubertal signs (yr)**	Thelarche at 0.7	Thelarche at 0.7	Menarche at 8.5	Thelarche at 5 Pubarche at 7.5	Thelarche and pubarche at 5.9	Thelarche at 7.6	Thelarche at 6.7
**At the time of diagnosis**			***a***		***b***		
**CA (yr)**	1.2	1.2	-	9.7	5.9	7.9	6.7
**Tanner stage**	2	2	-	4	2	2	2
**Height SDS**	-1.7	-1.8	-	-0.1	1.5	2.6	0.8
**BMI SDS**	-0.15	-0.17	-	1.5	1.3	1.5	-0.7
**Bone age (yr)**	-	-	-	12	8.5	10	9.7
**Basal LH (IU/L)^*c*^**	0.7	<0.1	-	< 0.6	0.36	0.2	4.1
**Peak LH (IU/L)^*c*^**	6.8	4.9	-	13.2	2.38	23.4	18
**Basal FSH (IU/L)^*c*^**	2.8	2.2	-	1.7	2.3	1.7	5.5
**Peak FSH (IU/L)^*c*^**	86.1	33.1	-	13.5	18.4	17.6	10
**Estradiol (pg/mL)^*c*^**	<11.5	<11.5	-	< 13	23.1	5	72
**Family history of CPP**	Monozygotic twin sister with CPP	Monozygotic twin sister with CPP	No	No	No	No	No
**Neurocognitive phenotypes**	Microcephaly ^***d***^ Speech therapy and mild difficulties in literacy	Microcephaly ^***d***^	Autism Macrocephaly ^***e***^	No	No	No	No
**Other clinical features**	Subtle dysmorphisms: short neck, thin upper lip, up slanting palpebral fissures	Subtle dysmorphisms: short neck, thin upper lip, up slanting palpebral fissures	At childhood: Hyperphagia with weight gain At adolescence: Obesity	At early adulthood: Short stature Irregular cycles Polycystic ovary (at ultrasound)	At adolescence: Irregular cycles Hirsutism Biochemical hyperandrogenism	No	SGA ^***f***^ Birth weight SDS -2.6 Birth length SDS -4.2

CPP: central precocious puberty; CA: chronological age; SDS: standard deviation score; BMI: body mass index; LH: luteinizing hormone; FSH: follicle-stimulating hormone; NA: not available; SGA: small for gestational age

a Patient 3 was first referred to the Endocrinology unit at 11.6 years, reporting precocious menarche at 8.5 years with subsequent regular menstrual cycles, corresponding to a diagnosis of CPP.

b Patient 5 presented for evaluation at 14 years with clinical and biochemical hyperandrogenism. She had undergone endocrine investigation in another institution at 5.9 years for a presentation of pubarche and thelarche. No management was instituted, and she was lost to follow-up. Afterwards, she presented with menarche at 12 years. There are no hormonal data available confirming the exact age at central puberty onset.

c LH, FSH, and estradiol levels were measured by electrochemiluminescence assay. Hormonal criteria for confirming CPP were pubertal levels of basal and/or GnRH-stimulated LH. Cut-off values were ≥ 0.3 IU/L for basal LH and > 5.0 IU/L for GnRH-stimulated LH peak. Pre-pubertal ranges were < 4 UI/L for basal FSH and < 15 pg/mL for estradiol.

d Head circumference on the 2^nd^ percentile.

e Head circumference on the 97.5^th^ percentile.

f Small for gestational age was defined by birth weight and/or birth length ≤ -2.0 standard deviation score for gestational age, according to the Usher & McLean method.

**Table 3:** Comparison of allele frequencies of rare potentially damaging *MECP2* variants between girls with central precocious puberty (CPP) from the current study (cases) and females from the gnomAD public database (controls) ^*a*^.

Groups	Number of potentially damaging alleles ^*b*^	Total number of alleles evaluated	Allele frequency (%)	Unadjusted *p*-value^*c*^
**CPP girls (n=383)**	7	766	0.91	1.587e-05
**Females from the gnomAD (n=35,750)**	69	71,500	0.09	

a Data from the gnomAD were extracted from the v3.1.2 dataset (GRCh38), containing data from genomes of diverse ancestries. The comparison between cases and controls included only females, since males are hemizygous for the *MECP2* gene and there was no *MECP2* variant identified in CPP boys from the current study.

b Potentially damaging *MECP2* variants were defined as rare variants (MAF ≥ 0.01%) that were categorized as pathogenic, likely pathogenic, or variant of uncertain significance by ACMG criteria.

c Statistical significance was set at *p*-value < 0.05.

## Data Availability

Anonymised data will be made available upon reasonable request for academic use and within the limitations of the informed consent. Proposals should be made to the corresponding author (anacl@usp.br). Data will be shared according to the data protection regulations of each hospital and country participating in the study.

## Research in context

### Evidence before this study

Precocious puberty is an endocrine disorder that affects children worldwide. In clinical practice, non-treated precocious puberty can lead to adverse outcomes in adulthood including short stature and psychosocial distress. In large population studies, early puberty timing has also been associated with diverse health conditions, such as obesity, type 2 diabetes, hypertension, cardiovascular disease, and cancer risk (breast and endometrial cancers in women and prostate cancer in men). The most common mechanism underlying precocious puberty is the premature reactivation of pulsatile hypothalamic gonadotropin-releasing hormone (GnRH) secretion, known as central precocious puberty (CPP). The recognition of factors and pathways involved in CPP has contributed to the understanding of the mechanisms involved in normal and abnormal GnRH reactivation. Recent advances based on precise clinical evaluation and high-throughput genetic approaches have allowed the identification of genetic etiologies of CPP. To date, loss-of-function mutations in two autosomal imprinted genes [Makorin ring finger protein 3 (*MKRN3*) and Delta like non-canonical Notch ligand 1 (*DLK1*)] are the most prevalent known monogenic causes of familial CPP, with epigenetic regulation of upstream pathways of GnRH neuronal activity implicated in the pathogenic mechanisms of the deficiency of both genes. Nevertheless, new players involved in premature pubertal onset are to be elucidated in individuals with idiopathic CPP.

Methyl-CpG-binding protein 2 (*MECP2*) is a X-linked gene (chromosome Xq28) which encodes a protein capable of binding to methylated DNA in promoter regions, functioning as a repressor or an activator of gene transcription. In humans, loss-of-function mutations in *MECP2* are usually associated with neurodevelopmental conditions, mainly with a severe disorder known as Rett syndrome. Early pubertal development has been documented among individuals with Rett syndrome due to *MECP2* mutations, including clinical studies of large Rett cohorts with mean age of puberty onset considerably earlier than healthy individuals. Despite this, *MECP2* defects have not been evaluated in individuals with idiopathic CPP to date.

### Added value of this study

To our knowledge, this is the first clinical research to investigate whether MECP2 biology is associated with an idiopathic CPP phenotype, evaluating its potential role in the etiology of this endocrine disorder. We identified rare heterozygous likely damaging variants in the *MECP2* gene in multiple unrelated girls with sporadic CPP, with or without mild neurodevelopmental abnormalities, revealing a potential X-linked form of premature pubertal development. *MECP2* emerged as a potential player in the hypothalamic control of human pubertal timing, thus further increasing the evidence of (epi)genetic mechanisms in this biological process.

### Implications of all the available evidence

Our findings provided basis for the investigation of potential genetic factors involved in the etiology of CPP in future studies. This strategy may allow individuals with CPP to benefit from a precision medicine approach, providing them more personalized strategies of follow-up with increased long-term clinical surveillance. In addition, the (epi)genetic discoveries involving the etiology of CPP may contribute to potential new treatment targets in the future.
