# Supplementary material for: Rare Variants in the MECP2 Gene in Girls with Central Precocious Puberty
Source: Lancet Diabetes Endocrinol. Author manuscript; Available in PMC 2023 Sep 13. (PMC7615084; doi:10.1016/S2213-8587(23)00131-6)
Supplement: Supplementary Material [file EMS187253-supplement-Supplementary_Material.pdf]

THE LANCET
Diabetes & Endocrinology

Supplementary appendix

This appendix formed part of the original submission and has been peer reviewed. We post it as supplied by the authors.

Supplement to: Canton APM, Tinano FR, Guasti L, et al. Rare variants in the *MECP2* gene in girls with central precocious puberty: a translational cohort study. *Lancet Diabetes Endocrinol* 2023 published online June 26. https://doi.org/10.1016/S2213-8587(23)00131-6.

Rare Variants in the *MECP2* Gene in Girls with Central Precocious Puberty: a translational cohort study

Supplementary Material

Table of Contents

Methods …………………………………………………………………………….. **2**
Patients ……………………………………………………………………........... 2
Genetic studies ……………………………………………..……………….......... 2
Exome sequencing …………………………………………………………...………... 3
Targeted sequencing ……………………………………………………………………… 3
High-throughput sequencing data analysis ……………………………….………... 3
Sanger sequencing ………………………………………………………………….... 3
Pathogenicity assessment of identified sequence variants ………………….………... 4
Microsatellite analysis …………...……………………………………...………... 5
Identification of parental origin of *de novo MECP2* variants ..…………….………….. 5
Chromosome X inactivation studies in blood samples .....……..………………….......... 5
Look-up of *MECP2* variants in UK Biobank ……………….……………… 6
Assays in mice ………………..………………………….………………………….. 6
Immunohistochemistry ……………………………………………………................... 7
Results ……………………………………………………………………………. **7**
MECP2 rare variants and clinical phenotypes ………………………..……….…..... 7
Patients 1 and 2 ………………………………………………………………............... 7
Patient 3 ……………………………………………………….................................. 9
Patient 4 ……………………………………………………….................................. 10
Patient 5 ……………………………………………………….................................. 11
Patient 6 ……………………………………………………….................................. 12
Patient 7 ……………………………………………………….................................. 13
Supplementary Table ……….……………………………………………………...... **14**
Table S1 ……………………………………………………………………………...... 14
References ………………………………………………………………….......... **15**

Methods

Patients

The diagnosis of CPP was defined by the development of progressive pubertal signs, primarily Tanner stage 2 breast development (thelarche) and testicular volume (≥ 4 mL) before age 8 years in girls and 9 years in boys, respectively (1). Basal and/or GnRH-stimulated luteinizing hormone (LH) levels within the pubertal range confirmed the diagnosis of central precocious puberty (CPP). For most patients, LH levels were measured by electrochemiluminescence assay. In this setting, cut-off values were ≥ 0.3 IU/L for basal LH in both sexes and > 5.0 IU/L for GnRH-stimulated LH peak in both sexes. For a subset of patients, LH levels were measured by immunofluorometric assay. For this methodology, cut-off values for basal LH were > 0.6 IU/L for basal LH in both sexes, while for GnRH-stimulated LH peak were > 6.9 IU/L for girls and > 9.6 IU/L for boys (1). Accelerated linear growth and accelerated bone maturation (> 1 year in relation to chronological age according to the Greulich and Pyle method) were additional criteria. In females, precocious menarche (≤ 9 years) indicated an equivalent clinical signal of CPP (2). All patients were clinically assessed by pediatric endocrinologists with expertise in genetic disorders. No patients had anatomical abnormalities related to CPP on magnetic resonance imaging (MRI) of the central nervous system (CNS). Additionally, all had genomic DNA previously sequenced for the coding regions of genes causing monogenic CPP (*MKRN3*, *DLK1*, *KISS1R,* and *KISS1*), ruling out mutations in these genes. Patients were classified as having familial CPP if they had at least one first-, second-, or third-degree affected relative. Female relatives were considered affected when they had documented CPP or precocious menarche (≤ 9 years). Male relatives were considered affected when they had documented CPP. Suggestive characteristics of precocious puberty in males were previous history of early full puberty (including full facial shaving and voice breaking) and early timing of growth spurt and growth completion. In addition, adult short stature was a suggestive characteristic of precocious puberty in both sexes (1, 2).

Genetic studies

In all individuals studied, genomic leukocyte DNA was extracted from whole blood samples using standard techniques (3).

Exome sequencing

Sixty-two cases (from 56 unrelated families) underwent exome sequencing, according to previously published protocols. Briefly, the libraries were constructed with the SureSelect Target Enrichment system (Agilent Technologies, CA). The sequences were generated in the Illumina HiSeq 2500 platform running on paired-end mode. The exome data were filtered for loss-of-function and nonsynonymous variants present in a heterozygous state in the proband and based on the family pedigree.

Targeted sequencing

Seventy-one cases were submitted to a customized panel of targeted sequencing, which included the Methyl-CPG-binding protein 2 (*MECP2*) gene (SureDesign tool, Agilent Technologies, CA). The panel was designed with 746 genes known or candidates to be associated with developmental, endocrine, and metabolic disorders. The enriched DNA libraries were sequenced in NextSeq 500 platform on paired-end mode (Illumina, CA).

High-throughput sequencing data analysis

The exome and the targeted sequencing data were screened for rare variants (minor allele frequency ≤ 0.01% in public and in-house databases) located in exonic regions and consensus splice site sequences. The variant filtration prioritized genes based on their potential to be pathogenic: loss-of-function variants and variants predicted to be pathogenic by multiple *in silico* programs. The sequencing reads carrying candidate variants were inspected visually using the Integrative Genomics Viewer (IGV; Broad Institute, Cambridge, MA).

Sanger sequencing

The remaining 271 patients were screened for allelic variants in *MECP2* by Sanger sequencing. Furthermore, putative damaging variants observed by exome and targeted panel sequencing, as well as familial segregation analysis were validated and genotyped also by Sanger sequencing, as appropriate. The primers for *MECP2* sequencing were designed to amplify all exons and exon-intron boundaries of the NM_001110792.2 transcript (primers sequence and amplification

protocols are available upon request). The PCR amplification was followed by sequencing of the products with the use of the conventional Sanger method, as previously described.

Pathogenicity assessment of identified sequence variants

All possible candidate sequence variants were classified according to the American College of Medical Genetics and Genomics (ACMG) standards with five categories of pathogenicity: pathogenic, likely pathogenic, variant of uncertain significance (VUS), likely benign, and benign (4). The population data included two public genomic databases: the Genome Aggregation Database (gnomAD, https://gnomad.broadinstitute.org) (5) and the Online Archive of Brazilian Mutations (ABraOM, https://abraom.ib.usp.br) (6). Variants were also evaluated in an in-house exome database of a local cohort of 523 unrelated individuals with the same ethnic background from the School of Medicine of the University of Sao Paulo (SELA laboratory, https://intranet.fm.usp.br/sela/pt-br/) (7). Possible candidate sequence variants were evaluated for pathogenicity prediction through several different computational tools, including CADD, PolyPhen2, SIFT, REVEL, MetaLR, MetaRNN, MetaSVM, Mutation Assessor, Mutation Taster, FATHMM, FATHMM-MKL, LRT, M-CAP, and PROVEAN (https://varsome.com; https://franklin.genoox.com/clinical-db/home). Furthermore, a function-structure analysis of variants was performed. For this purpose, the experimental structures of MECP2 alone or in complex with DNA were extracted from the ProteinDatabank (8), whereas the three-dimensional model of MECP2 was obtained from the AlphaFold database (https://alphafold.ebi.ac.uk). In addition, computational protein modelling analysis was performed for missense variants using Yasara software (Vienna, Austria) with FoldX toolsuite, which provided an analysis of their effect of on the stability, folding and dynamics of protein (9). Candidate variants were evaluated for submissions in 1) the international literature, 2) the ClinVar, a public archive of human variations and related phenotypes (https://www.ncbi.nlm.nih.gov/clinvar/) and 3) the RettBASE, a database to curate mutation data related to Rett syndrome, including pathogenicity assessment of a large number of *MECP2* mutations (http://mecp2.chw.edu.au/index.shtml).

To provide further genetic evidence of the association of *MECP2* with CPP phenotype, a collapsed analysis of variants across the entire gene was performed, comparing allele frequencies

between our cohort of CPP patients (cases) and the Genome Aggregation Database (gnomAD) public database (controls). The gnomAD database is composed of exome and genome sequences from around the world that were reprocessed through equivalent pipelines. The samples from the CPP cohort and the gnomAD database were not jointly sequenced or called. We extracted data from the gnomAD v3.1.2 dataset (GRCh38), which contains data from genomes of diverse ancestries. As *MECP2* is an X-linked gene, males are hemizygous for the gene. Therefore, the comparison between cases and controls was separated by sex. In the gnomAD dataset, we selected *MECP2* variants in females with similar characteristics to the *MECP2* variants observed in the girls from the cohort of CPP: rare variants (MAF ≤ 0.01%) that were categorized as pathogenic, likely pathogenic, or variant of uncertain significance. Allele frequency differences between groups were analyzed by Fisher´s exact test. Statistical significance was set at *p*-value <0.05. Statistical analyses were performed using R studio (R studio 2022.120+353).

Microsatellite analysis

When necessary, biological paternity was confirmed by the AmpFlSTR® Identifiler® PCR Amplification Kit, a short tandem repeat multiplex assay that amplifies 15 tetranucleotide repeat *loci* and the Amelogenin gender-determining marker using manufacture instruction (Life Technologies, Carlsbad, CA).

Identification of parental origin of de novo MECP2 variants

Studies to determine parental origin of variants were performed in patients carrying *de novo MECP2* variants by analysis of linkage between the *MECP2* variants and polymorphisms (10). We searched for heterozygous inherited polymorphisms near the *MECP2* variants in sequencing studies of patients carrying *de novo* variants. The inheritance of the heterozygous polymorphism was then established (maternal or paternal). TA cloning was performed to establish the allele separation (Thermo Fisher Scientific, Massachusetts, USA). A second round of PCR with the cloning products (cloning DNA in vector) was performed to evidence the inheritance of the X-chromosome carrying the *MECP2* variant and near heterozygous polymorphism.

X-chromosome inactivation (XCI) analysis in blood samples

The XCI status was estimated by assessing the X active/X inactive ratios in genomic DNA samples from peripheral blood using methylation sensitive restriction enzyme. The evaluation was based on the presence of microsatellite polymorphisms in the *AR* (11) or *RP2* genes (12). Microsatellites were analysed by capillary electrophoresis (ABI 3730 DNA Analyzer, Thermo Fisher Inc.) and using the GeneMarker™ software (SoftGenetics). The XCI ratios were estimated according to Bittel *et al* (13). The XCI ratios below 80-20% were considered to represent a random pattern; between 80-89%, moderate skewing, and >90-10%, extreme skewing. A skewing >90-10% is often considered clinically relevant (14). Sufficient DNA was not available to study two individuals with *MECP2* defects: the carrier mother (I.2) from patient 4 and the index (II.1) case patient 6.

Look-up of MECP2 variants in UK Biobank

We evaluated the effect of *MECP2* variants in the UK Biobank (UKBB; https://www.ukbiobank.ac.uk), currently the largest population study worldwide of ~420,000 research participants with exome sequencing and broad phenotypic characterization. Study participant and variant quality control as well as variant annotation were performed as described in Gardner *et al* (15). We performed a look-up of the four identified *MECP2* variants in recalled age at menarche (AAM) whole exome sequencing data within the UKBB. AAM was derived using data from field 2714. For individuals with missing data at each recorded instance, data from the next recorded instance was used if available. In total, data on 222,283 women on European genetic ancestry were analyzed and 1,204 *MECP2* variants were present in the sample. Similarly, we also performed a look-up of the four identified *MECP2* variants in recalled time at voice breaking in men within the Biobank. In the UKBB, voice breaking in men was recalled in three categories: younger than his peers, about average age, and older than his peers. Additionally, in the UKBB population, we searched for the high-confidence protein-truncating variants in *MECP2* gene and tested an association of this functional category of variant with traits considered as related to puberty. The phenotypes evaluated were age at menarche, voice breaking, BMI, height, and testosterone concentrations.

Assays in mice

Immunohistochemistry

Mice were collected from timed crosses of C57BL/6 mice. Pubertal timing was assessed by vaginal opening, a proxy of pubertal onset in female mice. Tissues were fixed in 4% paraformaldehyde (PFA) in PBS, cryoprotected in 30% sucrose, and frozen in OCT compound (VWR). 16-µm thick serial coronal sections were collected free floating in PBS. Sections were incubated with primary antibodies (anti-MECP2, Abcam; anti-GnRH, Immunostar;) diluted 1:200 or 1:1000 respectively in PBS-Triton 0.1%, overnight at room temperature (RT) as used in (16). After three washes with PBS-Triton 0.1%, slides were, for immunohistochemistry, incubated for 2 hours at RT with biotin-conjugated goat secondary antibodies (Vector Laboratories), diluted 1:300 in PBS and, after further washes, with the avidin-biotin complex (ABC staining kit, Vector Laboratories). The sections were reacted with 3,3'-diaminobenzidine (DAB, Vector Laboratories) and mounted in an aqueous compound formed by PBS and glycerol (3:1). For immunofluorescence sections were incubated for 2 hours at RT with secondary antibodies (488-conjugated goat anti-mouse and 568-conjugated goat anti-rabbit Fab fragments (1:500; Jackson Immunoresearch), and then reacted with 4',6'-diamino-2-phenylindole (DAPI, Vector Laboratories) and mounted on Superfrost Plus slides (VWR). Images were acquired using a Leica DM5500B microscope (Leica, Nussloch, Germany), equipped with a DCF295 camera (Leica) and DCViewer software (Leica), and then processed with Abode Photoshop CS6 and Adobe Illustrator CS6.

Results

It is established that the *MECP2* gene consists of four exons and, due to alternate splicing of exon 2, it encodes two isoforms (MECP2E1 and MECP2E2) differing in their N-terminal sequences (17, 18). The variants identified in the current study were within the transcript (NM_001110792.2) corresponding to the MECP2E1. Notably, distinct studies showed the expression of MECP2E1 in the brain and its putative role in the developing brain (19, 20).

***MECP2* rare variants and clinical phenotypes**

Patients 1 and 2

The heterozygous missense variant c.289C>T, p.Arg97Cys in *MECP2* was identified in two monozygotic twin sisters of a family of European ancestry from the United Kingdom (Table 1). Patient 1 presented with very early thelarche at 0.7 years and subsequently displayed pubertal basal and GnRH-stimulated LH levels at 1.2 years (Table 2), since when she has been treated with long-acting GnRH analog. Patient 2 presented with the same clinical signs, whilst her GnRH-stimulated LH peak measurement at 1.2 years was just below the biochemical threshold of 5 IU/L for diagnosis of CPP. In view of her clinical phenotype and of her monozygotic twin diagnosis of CPP, treatment with long-acting GnRH analogue was initiated concurrently with her sister. Moreover, at 1.2 years, both girls had pelvic ultrasound findings suggestive of precocious puberty due to an increased uterus and ovaries volumes for age. Thereafter, both girls showed signs of progressive CPP, showing ongoing raised serum LH concentrations and high growth velocity, despite ongoing treatment. This led to the shortening of the dose interval of long-acting GnRH analogue in both girls at 2.1 years and at 5.7 years. To date, Patients 1 and 2 are still under GnRH analogues treatment at 10 years old. Both twin sisters had microcephaly (head circumference on the 2nd percentile) and subtle dysmorphic features, including short neck, thin upper lip, and up-slanting palpebral fissures. They had met the milestones of neuro-psychomotor development within the range of healthy children with no subsequent loss of acquired skills. Both girls were followed by clinical geneticists, who did not identify clinical criteria for the diagnosis of a recognized syndrome. They attended mainstream school; Patient 1 had speech therapy and additional help with literacy. They were children of non-consanguineous parents (individuals I.1 and I.2), who had normal pubertal development. Similarly, there was no report of precocious puberty in other family members. Familial segregation analysis showed this was a *de novo* variant. Arginine 97 is located at the Methyl-CpG-binding domain, one of the main functional domains of MECP2 protein primarily associated with methylated DNA binding (18). The variant is located at a highly conserved region among species, and it was predicted to be damaging in the following 19 (of 21) *in silico* programs: REVEL (score 0.6869), MetaLR, MetaRNN, Meta SVM, BayesDel addAF, BayesDel noAF, DANN, Deogen2, FATHMM, FATHMM-MKL, LIST-S2, LRT, M-CAP, MVP, MutationTaster, PROVEAN, PrimateAI, SIFT, SIFT4G. According to the stability

analysis, the variant was identified as highly destabilizing to the protein. This missense variant has not been reported previously in the literature or in RettBASE. Meanwhile, it had two clinical testing submissions in ClinVar: 1) as a variant of uncertain significance and 2) as a likely pathogenic variant associated with Rett syndrome phenotype in one individual with motor and speech delay, intellectual disability, seizure, and cardiomyopathy.

The standard X-inactivation analysis performed in leukocyte genomic DNA of Patients 1 and 2 identified an extreme skewing in Patient 1 (Supplementary Table 1). This finding implied that one of the X-chromosomes was preferentially inactivated in this patient, at least in the blood. No RNA sample from Patient 1 was available to perform further allele-specific expression analysis that could clarify which X-chromosome was preferentially inactivated (the mutant or the wild-type).

Patient 3

A novel missense variant, c.528C>A, p.Ser176Arg, was identified in Patient 3, a Caucasian Brazilian girl with a sporadic clinical picture including precocious menarche (Table 1). This patient was first referred to the Endocrinology unit at 11.6 years for a history of hyperphagia followed by progressive weight gain during childhood reaching obesity at adolescence. Endocrinological evaluation elicited that she had precocious menarche at 8.5 years with subsequent regular menstrual cycles, indicating the diagnosis of CPP (Table 2). Notably, she also displayed autistic behaviors (with stereotyped body movements) and macrocephaly (head circumference 97.5th percentile). Standardized Neuropsychological evaluation (using the Wechsler Intelligence Scale for Children Test, the Children's Apperception Test, and the House-Three-Person Test) during childhood showed that her global intellectual capacity was within the average range. However, she had variability in specific functions, with greater difficulty in verbal activities and limitations in coordination, writing, expressiveness, and social situations. She had serial electroencephalograms (EEGs) during childhood. Between 8 and 9 years old, there were alterations suggestive of epilepsy on her EEG, which did not appear in later EEGs. To date, she had no clinical manifestations of seizures. She reached an adult height SDS of -1.4, at the lower limit of her target height (target height SDS -0.6). The patient was followed by a multidisciplinary

team, including clinical geneticists and psychiatrists. Nevertheless, she did not have clinical criteria for the diagnosis of a recognized syndrome. She was born at term by cesarian delivery due to the presence of meconium, but adequate growth for gestational age (birth weight SDS 0.4 and birth length SDS 0.2). She was the first child of non-consanguineous parents. There was no history of premature pubertal signs among family members. Her father had normal puberty and adult height SDS -1.3; her mother had menarche at 13 years and adult height SDS 0.3; and her younger sister had menarche at 11 years and height SDS 0.2. Familial segregation analysis showed this was a *de novo* variant. Parental origin studies evidenced that the p.Ser176Arg variant arose on the paternally derived X-chromosome. Serine 176 is located within the intervening domain of MECP2 and is highly conserved among species. The intervening domain has been related to structural and functional roles in the MECP2 protein (21). Serine 176 residue phosphorylation was identified as a post-transcriptional modification of MECP2 with impact on its DNA or chromatin binding properties, especially in neuronal development (22). The missense substitution was predicted to be damaging in 15 (of 20) *in silico* programs as follows: MetaLR, MetaRNN, MetaSVM, BayesDel addAF, BayesDel noAF, CADD (score 24.7), DANN, FATHMM, FATHMM-MKL, LRT, M-CAP, MVP, MutationTaster, Polyphen2, SIFT. According to the stability analysis, the variant was identified as highly destabilizing to the protein. To date, this variant has not been reported in the literature, in ClinVar, or in RettBASE.

Patient 4

A rare *indel* insertion, c.15_23dup, p.Ala6_Ala8dup, was identified in Patient 4, a Caucasian Brazilian girl with sporadic CPP (Table 1). She had thelarche since birth with progression from age 5 years, and pubarche at 7.5 years. She was first referred to the Sao Paulo University hospital at 9.7 years, presenting with Tanner stage 4 breast development and bone age advancement (bone age 12 years) (Table 2). At the time, she had pubertal LH levels, initiating treatment with a long-acting GnRH analogue. Neurological, behavioral, motor, or epileptic findings were not identified. Electroencephalogram showed no abnormalities. At adolescence, she developed irregular menstrual cycles and polycystic ovarian morphology on ultrasound. She reached an adult height SDS of -2.0, providing evidence of short stature (target height SDS -1.4). Her birth parameters

were not available. She was the second child of non-consanguineous parents. There was no report of precocious puberty among family members. Her father had adult height SDS -1.4, and her mother had adult height SDS -1.2 and menarche at 11 years.

Familial segregation analysis revealed that the p.Ala6_Ala8dup variant was inherited from her unaffected mother (individual I.2). Therefore, her mother was identified as an asymptomatic carrier female. Although uncommon, this pattern has been described in several cases of X-linked dominant disorders, including Rett syndrome due to *MECP2* mutations (23). The main mechanism suggested was that the asymptomatic carrier females could show a pattern of non-random X-inactivation (with the mutated *MECP2* allele being preferentially inactivated) and could escape phenotypic expression of the disorder (23). Peripheral leukocyte DNA from the mother of patient 4 (individual I.2) for the study of X chromosome inactivation was not available (Supplementary Table 1). In addition, the mechanism of X chromosome inactivation may have different patterns among different tissues, such as hypothalamic neurons, which could also influence the phenotypic expression of the disorder (14).

The p.Ala6_Ala8dup variant is located at the N-terminal domain, in a region considered to be highly conserved across mammalian species (17). The N-terminal domain has been implicated to have structural and functional roles in increasing the affinity of MECP2 for binding to DNA (18). The p.Ala6_Ala8dup results in the insertion of 3 amino-acids to the MECP2 protein, but otherwise preserves the integrity of the reading frame. To date, the p.Ala6_Ala8dup has been reported in two patients (of 754) with mild intellectual disability in a study evaluating a possible association of variants in exon 1 of *MECP2* with neurocognitive phenotypes other than Rett syndrome (17). Pubertal development was not assessed in this cohort. ClinVar and RettBASE reported this insertion in correspondence to this previous study (17). Additionally, ClinVar had two clinical testing submissions of this variant, where it was considered as of uncertain significance for association with neurocognitive phenotypes other than Rett syndrome.

Patient 5

The rare *indel* insertion c.15_23dup, p.Ala6_Ala8dup was also identified in Patient 5, a Brazilian girl (Table 1). Patient 5 presented for evaluation at 14 years with irregular cycles (amenorrhea),

hirsutism, and biochemical hyperandrogenism. As a child, she had undergone endocrinological investigation in another institution due to pubarche and thelarche at 5.9 years associated with bone age advancement (bone age at 8.5 years) and growth acceleration (height SDS 1.5) (Table 2). No treatment was instituted, and she was lost to follow-up. Afterwards, she presented with menarche at 12 years. There are no hormonal data available confirming the exact age at which LH reached pubertal levels. Neurological, behavioral, or motor findings were not identified. She was the only child of non-consanguineous parents and born at term by cesarian delivery with adequate growth for gestational age (birth weight SDS 0.3). There was no report of precocious puberty among family members. Familial segregation analysis identified that the insertion was not inherited from her unaffected mother; however, no clinical data or DNA from the father were available. Genetic findings regarding the p.Ala6_Ala8dup variant are mentioned above in the description of Patient 4.

Patient 6

A novel rare heterozygous insertion in the 3'untranslated region (UTR) of *MECP2*, c.*36_*37insT, was identified in a girl of a family of European ancestry from Spain. She had thelarche at 7.6 years. At 7.9 years, she presented for her first endocrine visit with pubertal LH levels, height SDS at 2.6, and bone age advancement (bone age 10 years) (Table 2) since which time she was treated with a long-acting GnRH analog. No neurological, behavioral, motor, or developmental abnormalities were identified. There was no report of precocious puberty among family members. Familial segregation analysis identified that the insertion was inherited from her apparently unaffected mother. Therefore, the inheritance pattern of *MECP2* variant in Patient 6 seemed to be similar to that of Patient 4, and the same mechanisms above mentioned could to be considered. Peripheral leukocyte DNA from Patient 6 (individual II.1) for the study of X chromosome inactivation was not available (Supplementary Table 1).

The 3'UTR position of the variant is highly conserved across mammals. The variant was not identified in the public database gnomAD or ABraOM. Moreover, it was not previously described in publications, in ClinVar, or in RettBASE. Notably, *MECP2* 3'UTR harbors a large number of polyadenylation sites and miRNA binding sites demonstrated to regulate MECP2 mRNA

maturation and stability, respectively (24). Applying the ACMG criteria, the c.*36_*37insT was considered as a variant of uncertain significance.

Patient 7

The rare 3'UTR insertion c.*36_*37insT was also identified in a heterozygous state in Patient 7, a girl of another family of European ancestry from Spain. She developed thelarche at 6.7 years of age, when she presented for the first endocrine visit. The findings of pubertal LH levels and bone age advancement (bone age 9.7 years) confirmed the diagnosis of CPP. No neurological, behavioral, developmental, or motor anomalies were reported. She was born at term and was small for gestational age for weight (birth weight SDS -2.6) and for length (birth length SDS -4.2). The proband was the only child of non-consanguineous parents. There was no history of CPP among family members. Her father had adult height SDS 1.1, and her mother had adult height SDS 0.0 and menarche at 12 years. Familial segregation analysis identified that this was a *de novo* variant. Genetic findings about the 3'UTR insertion c.*36_*37insT are as described above for Patient 6.

Supplementary Table 1. Analysis of X-chromosome inactivation (XCI) patterns in available blood samples of females with CPP carrying *MECP2* variants and their mothers.

Pedigree ID	Status	XCI ratio (%)	XCI status
Patients 1 and 2 (p.Arg97Cys)			
**II.1**	Index	100:0	Extreme skewing
**II.2**	Index	60:40	Random
Patient 3 (p.Ser176Arg)			
**I.2**	Mother with normal puberty	59:41	Random
**II.1**	Index	66:34	Random
Patient 4 (p.Ala6_Ala8dup)			
**I.2**	Carrier mother with normal puberty	NA	NA
**II.1**	Index	52:48	Random
Patient 5 (p.Ala6_Ala8dup)			
**I.2**	Mother with normal puberty	44:56	Random
**II.1**	Index	40:60	Random
Patient 6 (c.36*_37*insT)			
**I.2**	Carrier mother with normal puberty	42:58	Random
**II.1**	Index	NA	NA
Patient 7 (c.36*_37*insT)			
**I.2**	Mother with normal puberty	60:40	Random
**II.1**	Index	84:16	Moderate skewing

NA: DNA not available

References:

1. Latronico AC, Brito VN, Carel JC. Causes, diagnosis, and treatment of central precocious puberty. Lancet Diabetes Endocrinol. 2016;4(3):265-74.
2. Canton APM, Krepischi ACV, Montenegro LR, et al. Insights from the genetic characterization of central precocious puberty associated with multiple anomalies. Hum Reprod. 2021;36(2):506-18.
3. Miller SA, Dykes DD, Polesky HF. A simple salting out procedure for extracting DNA from human nucleated cells. Nucleic Acids Res. 1988;16(3):1215.
4. Richards S, Aziz N, Bale S, et al. Standards and guidelines for the interpretation of sequence variants: a joint consensus recommendation of the American College of Medical Genetics and Genomics and the Association for Molecular Pathology. Genet Med. 2015;17(5):405-24.
5. Karczewski KJ, Francioli LC, Tiao G, et al. The mutational constraint spectrum quantified from variation in 141,456 humans. Nature. 2020;581(7809):434-43.
6. Naslavsky MS, Yamamoto GL, de Almeida TF, et al. Exomic variants of an elderly cohort of Brazilians in the ABraOM database. Hum Mutat. 2017;38(7):751-63.
7. Lerario AM, Mohan DR, Montenegro LR, et al. SELAdb: A database of exonic variants in a Brazilian population referred to a quaternary medical center in São Paulo. Clinics (Sao Paulo). 2020;75:e1913.
8. Berman HM, Westbrook J, Feng Z, et al. The Protein Data Bank. Nucleic Acids Res. 2000;28(1):235-42.
9. Guerois R, Nielsen JE, Serrano L. Predicting changes in the stability of proteins and protein complexes: a study of more than 1000 mutations. J Mol Biol. 2002;320(2):369-87.
10. Trappe R, Laccone F, Cobilanschi J, et al. MECP2 mutations in sporadic cases of Rett syndrome are almost exclusively of paternal origin. Am J Hum Genet. 2001;68(5):1093-101.
11. Allen RC, Zoghbi HY, Moseley AB, Rosenblatt HM, Belmont JW. Methylation of HpaII and HhaI sites near the polymorphic CAG repeat in the human androgen-receptor gene correlates with X chromosome inactivation. Am J Hum Genet. 1992;51(6):1229-39.
12. Machado FB, Faria MA, Lovatel VL, et al. 5meCpG epigenetic marks neighboring a primate-conserved core promoter short tandem repeat indicate X-chromosome inactivation. PLoS One. 2014;9(7):e103714.
13. Bittel DC, Theodoro MF, Kibiryeva N, Fischer W, Talebizadeh Z, Butler MG. Comparison of X-chromosome inactivation patterns in multiple tissues from human females. J Med Genet. 2008;45(5):309-13.
14. Fieremans N, Van Esch H, Holvoet M, et al. Identification of Intellectual Disability Genes in Female Patients with a Skewed X-Inactivation Pattern. Hum Mutat. 2016;37(8):804-11.
15. Gardner EJ, Kentistou KA, Stankovic S, et al. Damaging missense variants in IGF1R implicate a role for IGF-1 resistance in the etiology of type 2 diabetes. Cell Genomics2022.
16. Cariboni A, Davidson K, Dozio E, et al. VEGF signalling controls GnRH neuron survival via NRP1 independently of KDR and blood vessels. Development. 2011;138(17):3723-33.
17. Harvey CG, Menon SD, Stachowiak B, et al. Sequence variants within exon 1 of MECP2 occur in females with mental retardation. Am J Med Genet B Neuropsychiatr Genet. 2007;144B(3):355-60.
18. Zachariah RM, Rastegar M. Linking epigenetics to human disease and Rett syndrome: the emerging novel and challenging concepts in MeCP2 research. Neural Plast. 2012;2012:415825.

19. Martínez de Paz A, Khajavi L, Martin H, et al. MeCP2-E1 isoform is a dynamically expressed, weakly DNA-bound protein with different protein and DNA interactions compared to MeCP2-E2. Epigenetics Chromatin. 2019;12(1):63.
20. Good KV, Vincent JB, Ausió J. MeCP2: The Genetic Driver of Rett Syndrome Epigenetics. Front Genet. 2021;12:620859.
21. Claveria-Gimeno R, Lanuza PM, Morales-Chueca I, et al. The intervening domain from MeCP2 enhances the DNA affinity of the methyl binding domain and provides an independent DNA interaction site. Sci Rep. 2017;7:41635.
22. Stefanelli G, Gandaglia A, Costa M, et al. Brain phosphorylation of MeCP2 at serine 164 is developmentally regulated and globally alters its chromatin association. Sci Rep. 2016;6:28295.
23. Hoffbuhr KC, Moses LM, Jerdonek MA, Naidu S, Hoffman EP. Associations between MeCP2 mutations, X-chromosome inactivation, and phenotype. Ment Retard Dev Disabil Res Rev. 2002;8(2):99-105.
24. Rodrigues DC, Mufteev M, Ellis J. Regulation, diversity and function of MECP2 exon and 3'UTR isoforms. Hum Mol Genet. 2020;29(R1):R89-R99.
